# All in My Head: Beckett, Schizophrenia and the Self

**DOI:** 10.1007/s10912-016-9384-6

**Published:** 2016-03-14

**Authors:** Elizabeth Barry

**Affiliations:** Department of English and Comparative Literary Studies, University of Warwick, Coventry, CV4 7AL UK

**Keywords:** Samuel Beckett, Schizophrenia, Philosophy, Psychiatry, Eugene Minkowski, Maurice Merleau-Ponty

## Abstract

This article will explore the representation of certain mental and somatic phenomena in Beckett’s trilogy of novels *Molloy*, *Malone Dies* and *The Unnamable*, exploring how his understanding of schizophrenia and psychosis informs his representation of the relationship between mind and body. It will also examine recent phenomenological and philosophical accounts of schizophrenia (Louis Sass, Josef Parnas, Shaun Gallagher) that see the condition as a disorder of selfhood and concentrate in it on the disruption to *ipseity*, a fundamental and pre-reflective awareness of self that leads to a loss of ‘grip’ (in the term of Merleau-Ponty) on concepts and percepts. Beckett’s writing might, it is argued, make such disruptions more tangible and intelligible. The article will also consider John Campbell’s argument that immunity of the first person to error—Sydney Shoemaker’s foundational philosophical idea that we cannot misspeak the first person pronoun—is revoked in states of psychosis, and relate such states to the moments in Beckett’s writing where this immunity is challenged, and quasi-psychotic experiences represented.

It has been frequently observed that there is a strong relationship between the language and sensibility of the work of writer Samuel Beckett and certain manifestations—both clinical and cultural—of the psychiatric disorder that is schizophrenia. Beckett is name-checked by Gilles Deleuze and Felix Guattari in their monumental 1972 work of cultural theory, *Anti-Oedipus*, his characters representative of what they call the ‘schizoid’ subject of late capitalism. His work is also a key test case for psychologist Louis Sass, who sees in it a schizophrenic sensibility shared by much modernist writing: a form of consciousness consistent with experiences of depersonalization, dissociation and hyperreflexivity (1992). Beckett scholars have also touched on the relationship, either observing a literal similarity between Beckett’s language and schizophrenic speech disorder (Coe 1983; Keatinge 2008) or seeing in his characters’ withdrawal from the ‘outer world’ and its materialist transactions what Richard Begam has called a “philosophical analogue” to the illness (1996, 45; Tahiri 2006). The persistence of the link between this poet-philosopher of modernity and the mental illness most often associated (metaphorically and literally) with the modern age has been both a contributing factor to and a reflection of the cultural capital the condition has accrued. The different kinds of identification between the condition of schizophrenia and both the form and content of Beckett’s work testify to the range and heterogeneity of meanings that the terms ‘schizophrenic’ and ‘schizoid’ have in cultural contexts. As Shane Weller has pointed out in his 2009 article on the idea of a ‘schizoid voice’ in Beckett’s work, there is little consensus about what the term ‘schizophrenia’ means among the Beckett critics who use it (33). Furthermore, this is not simply a matter of lexical nicety or over-eager theoretical analogy. There is no less debate in the clinical domain over the conceptual validity of the classification. The work that follows hopes to complement work in the philosophy of psychiatry engaging with this debate by offering, via a literary test case, a subjective perspective on experiences that could be seen to characterize schizophrenia—a perspective that points to underlying relationships between the affective aspects of the condition and its cognitive and perceptual features.

What claims can literature, and Beckett’s writing in particular, make in this regard? This paper contends that there is a correspondence between the sorts of mental phenomena (states of mind, beliefs, disordered perceptions of time, space and body) that Beckett describes and the experience of psychosis. It suggests that it might be fruitful for the student of Beckett to look at how both psychologists and philosophers describe what happens to linguistic self-reference under the pressure of psychotic states, descriptions that can be compared productively to those in Beckett’s mature work. Beckett’s work in its turn might help clinicians articulate ideas about the relationship between negative symptoms such as anhedonia and the perceptual deficits of attention, memory and time perception. To fully understand the founding condition of self-consciousness, the reflexive awareness which allows us to own our actions, thoughts and feelings and organize our perceptions and sensations but which eludes strictly materialist explanations, it may be helpful to employ literary and philosophical models alongside scientific ones. This paper will make a tentative exploration of these possibilities.

With these considerations in mind, the starting point for this discussion is not Louis Sass’s comment on Beckett himself, but his philosophically-informed psychology as it pertains to the experience of schizophrenia. In a 2003 article, ‘Schizophrenia, Consciousness and the Self’, Sass and his colleague Josef Parnas discuss what philosophers of mind call *ipseity*—the “vital and self-coinciding subject of experience or first-person perspective on the world” (428). It is no revelation to suggest that this subject position is often thematized in Beckett’s work in terms of threats to its continuation, threats similar in many cases to those encountered by the schizophrenic patient. And what is at stake in looking at the way in which psychotic states might threaten ipseity is what the philosopher Sydney Shoemaker called the “immunity principle”: the idea of self-reference “with immunity to the error of misidentifying the first-person pronoun” (1968). Shoemaker contends, following Wittgenstein, that in speaking of cognitive or experiential states (in Wittgenstein’s examples, ‘I try to lift my arm’, ‘I have toothache’, ‘I think it will rain’) we cannot misspeak the pronoun ‘I’; it has the authority of Sass’s vital and self-coinciding subjectivity (557). This, of course, is an immunity that Beckett’s work appears systematically to dismantle. From the trilogy of novels of the early 1950s, *Molloy, Malone Dies,* and the aforementioned *The Unnamable* (hereafter known as the Trilogy), onwards, his writing is correspondingly concerned with experiences akin to psychosis, a state that has been seen by John Campbell and others as offering a counter-example to the immunity principle (1999; Gallagher and Zahavi 2012, 211).

Even at this fundamental level, there are two dimensions to ipseity for both phenomenological philosophers such as Merleau-Ponty and Michel Henry and psychologists such as Sass and Shaun Gallagher (Merleau-Ponty 1989; Henry 1973; Sass and Parnas 2003; Gallagher 2000). There is “thematic, explicit or reflective intentionality” (Sass and Parnas 2003, 429), something akin to the rational *cogito*, and there is also a non-reflective, tacit sensibility (“operative intentionality” in Sass and Parnas’s terms) that constitutes our primary presence in the world. The latter can be identified with what Max Nordau termed *coenaesthesis* or the “organic dimly-conscious I” (1913, 249), the general sense of existence that comes from the sum of bodily feelings and operates at a level below consciousness proper. Beckett read about this concept in Nordau’s 1892 *Degeneration* and noted down both the term and this definition, as John Pilling and C. J. Ackerley have shown (Pilling 1999; Ackerley 2006),^1^ going on to use the term or its cognates in a number of his works, and appreciating particularly that the idea encompassed the intrauterine condition before birth with which he was abidingly concerned. As scholars have observed in recent years (Moorjani 2000; Oppenheim 2000; Salisbury 2008; Maude 2009), Beckett is arguably as interested in this kind of awareness, a world of sensation operating at a level of nervous integration in the brain below its cognitive processes, as he is with the “self-enclosed Cartesian theatre” with which early commentators associated him (Sass and Parnas 2003).

To start with, for the narrator of Beckett’s novel *The Unnamable*, the process of introspection seems promising:

How all becomes clear and simple when one opens an eye on the within, having of course previously exposed it to the without, in order to benefit by the contrast. I should be sorry, though exhausted personally, to abandon prematurely this rich vein.

He goes on in a more sceptical mode, however:

For I shall not come back to it in a hurry. But enough of this cursed first person, it is really too red a herring, I’ll get out of my depth if I’m not careful. But what then is the subject? Mahood? No, not yet. Worm? Even less. Bah, any old pronoun will do, providing one sees through it. Matter of habit. (1990, 354)

Beckett’s *Trilogy* becomes dominated by the imagining of situations where the immunity of the first person pronoun to error comes into question. Shoemaker argues that “the rules governing the use of this word determine once and for all what its reference is to be on any given occasion of its use, namely, that its reference is to the speaker, and leave no latitude to the speaker’s intentions in the determination of its reference” (1968, 559); Beckett’s narrator, however, relegates it to the status of any avatar, any passing character in his narrative.

A written ‘I’ is of course a different entity to a spoken ‘I’ and can be just as conventional, fictional or unreliable as any other pronoun or subject. Or almost. Even in an avowed work of fiction, there is a frisson of intimacy and an assumption of a certain privilege on the part of the reader in being addressed as ‘you’ and in being party to the directness that a first-person pronoun betokens. The relentless present tense of much of Beckett’s *Trilogy* appears at first to strengthen the proposed connection between the ‘I’ and some entity who was, at least, present when it was being written and able to determine its reference. Yet as this moment from *The Unnamable* shows, for the narrator, at least, it is when he as subject tries to look inside himself that the term ‘I’ becomes most inadequate and “cursed”. Saying I—in order to attribute to oneself certain beliefs, wishes, sensations, and perceptions—is never, as Shoemaker suggests it should be, “clear and simple”. The nature of the “within” is never elucidated, and the referent of the first person is, it is suggested, a matter of such uncertainty that one would get immediately “out of [one’s] depth” in hazarding its use. It is not—this is a point the narrator is firm on—that the subject is particularly complex; what the metaphorical “eye” of introspection sees, however, cannot be conveyed in language or connected to the most fundamental level of self-ascription.

There has been much comment on the way in which Beckett uses the idea of the ‘Not I’ although less acknowledgement that this was a term he first encountered in Nordau’s *Degeneration* (C. J. Ackerley 2006 being a notable exception). Nordau uses it to talk of a ‘self for others’ that he contrasts with the Cartesian self. Beckett, by contrast, reappropriates the term, using it in his work to refer to the impossibility of labelling and so ascribing consistency and agency to the self (a dilemma that finds most acute expression in his 1972 play *Not I*). It is illuminating, however, to look beyond the abstract labelling of person to the kinds of experience Beckett depicts in which ipseity is disturbed and the immunity of the pronoun ‘I’ to error challenged—experiences that echo those encountered in psychosis. For Sass, Gallagher, Henry and others, the fundamental feature of psychotic ipseity disturbance is hyperreflexivity, defined as forms of exaggerated self-consciousness in which a subject or agent experiences itself or something that would normally be inhabited as an aspect or feature of itself as a kind of external object (Sass 2000; Gallagher 2004; Henry 1973). This kind of altered consciousness is, I would argue, recognizable in Beckett’s trilogy, and the shorter French fiction (the novellas*, Texts for Nothing*), where not only a schizoid voice is actualized, in Weller’s terms, but a range of schizoid mental states as well. The ‘Not I’ cannot be explained directly, but it is related to such physical and perceptual experiences, dramatized in Beckett’s fiction and theatre, experiences which make the ontological uncertainty at work more tangible.

Beckett’s Molloy, the narrator of the first novel, feels in the term Beckett borrows from Nordau, “coenaesthetically speaking, […] more or less the same as usual” (1990, 54), his operative intentionality intact (more or less) for much of the work despite his growing infirmity and “terror-stricken” state. The same may not be said, however, for the protagonist of *Malone Dies*, the second novel of the *Trilogy*, who seems to experience something akin to psychotic hyperreflexivity. Sass and Parnas describe the phenomenon where “what might have been thought to be inalienable aspects of self come to seem separate or detached […] one’s arms or legs, one’s face, the feelings in the mouth or throat” (2003, 432). Malone’s body, likewise, becomes a series of objects which he can no longer govern and which he experiences as separate and external. His feet, for instance:

…my feet, which even in the ordinary way are so much further from me than all the rest, from my head, I mean, for that is where I am fled, my feet are leagues away. And to call them in, to be cleaned, for example, would I think take me over a month, exclusive of the time required to locate them. […] Is that what is known as having a foot in the grave?

This might be dismissed as a physiological problem, or a problem arising from damage to the local part of the sensorimotor map in his brain, were he not to go on:

And similarly for the rest. For a mere local phenomenon is something I would not have noticed, having been nothing but a series or rather a succession of local phenomena all my life, without any result. (235)

This speaks of an existing failure of moment-to-moment ipseity or sense of existence that also characterizes psychotic disturbance. Self-coincidence is compromised so completely that the narrative convention whereby the ‘I’ is simultaneously subject and object of a first-person narrative breaks down. Malone’s comment that “the subject falls far from the verb and the object lands somewhere in the void” (235) ostensibly describes what happens when he dozes off when writing and the wind turns the pages of his notebook, but seems to apply equally well to his disintegrating sense of personhood. The “exaggerated self-consciousness” characteristic of a disturbance of operative intentionality means, somewhat ironically, that self-presence cannot be achieved. Such passages, paving the way for the refusal of the first person itself in *The Unnamable*, illustrate what Sass identified in *Madness and Modernism* as a focus on hypertrophied self-consciousness common to both modernist writing and the schizophrenic patient.

Other features of psychotic experience reflected in Beckett’s work also go to the heart of the assumptions that surround a first-person fictional narrative. Part of the self-presence of *ipseity* consists in the tacit assumption of essential experiential differences between a remembered event and a remembered fantasy—the *noetic* aspect of consciousness. Shane Weller notes Beckett’s reading of Ernest Jones on hysteria in this respect, and his transcription of a passage (in his 1930s note-taking on psychology) about the vividness and saliency of fantasy for the hysteric to the point where it has an “equal significance to a real experience” (2009, 36). Weller identifies this idea with the late theatre (*Not I, Rockaby* and *Footfalls*), where the boundary between fantasy and reality “disintegrates” (36), but it also preoccupies Beckett from much earlier in his writing career. Beckett and his narrators are frequently concerned with their failure to distinguish remembered and imagined events and experiences. Molloy pictures how cows chew the cud, commenting “But perhaps I’m remembering things” as one might say, “But perhaps I’m imagining things” (9). Malone writes at one point, “You may say it’s all in my head”, a locution identifying memory with imagination: “You may say it is all in my head, and indeed sometimes it seems to me I am in a head and that these eight, no, six, these six planes that enclose me are of solid bone” (222). The experience he goes on to describe is itself suggestive of a hyperreflexive delusion whereby his skull is projected to the limits of his peripersonal space. The destabilizing of boundaries between external and internal, something the loss of noetic distinctions between the memory of objective reality and the imagination might bring about, is a condition that Beckett’s work makes audaciously literal.

Certain distortions of the subject’s ‘grip’ on the conceptual or perceptual field necessarily result from this condition, as the philosopher Maurice Merleau-Ponty described in his work on *The Phenomenology of Perception.* Perceptions of time and space are commonly distorted for the schizophrenic patient, as well as aspects of attention such as figure-ground segregation (Tsakanikos and Reed 2003). Beckett’s narrators also demonstrate such distortions of perception, which often compromise the immunity principle and so make difficult ascriptions of ownership to one’s sensations and states of mind. One feature of the failure of basic self-presence, for instance, is the loss of the aspect of operative intentionality whereby one is conscious in Sass and Parnas’s words of *something rather than something else*: the sharpness or stability with which figures or meanings emerge from and against some kind of background context (2003, 408). It is well known that Beckett’s early narrators lose this faculty, experiencing the world, as Neary does (after William James) in *Murphy* as a “big blooming buzzing confusion”– “ground mercifully free of figure”, as Beckett puts it in the terms of the Gestalt psychologists he was reading at the time (1993, 21; see also Salisbury 2010, 356–58).

A more concrete example of this has been observed by Ulrika Maude, writing about the link made in Nordau’s *Degeneration* between ‘hysteria’ (a category that encompassed certain kinds of psychosis at the time) and literal “blurred vision”, the hysterical or degenerate artist suffering from “trembling of the eyeball” who will “perceive the phenomena of nature trembling, restless, devoid of firm outline” (1913, 27). Beckett transcribed this passage in the reading notes he made in the 1930s, and it is likely to have been in his mind when he described the “confusion” in *Murphy*, and—as Maude points out—in the following passage from Beckett’s novella *The Calmative*, in which the images of the boy and goat that the narrator meets become confused to the point where “soon they were no more than a single blur which if I hadn’t known I might have taken for a centaur” (1995, 67; Maude 2013). The contexts for this “confusion” in Beckett’s work, encountered here as Nordau’s “blurring”, but elsewhere as aural “buzzing”, usually also encompass generalized problems with attributing salience to what is seen or heard, as well as with social interaction and affect—all also symptoms found together in those with schizophrenia. Perceptual difficulties arise consistently in Beckett’s work and are not just a feature of a weakness of the physical organs of sensory perception but symptomatic of some general loss of understanding of, or investment in, the organization of the world.

This loss of salience has a fuller treatment in the *Trilogy*, where the faculties of perception are also impaired, something directly implicated in the loss of distinction between world and self. For Malone, the “same old noises” have “merged into a single noise, so that all I heard was one vast continuous buzzing […] The noises of nature, of mankind and *even my own*, were all jumbled together in one and the same unbridled gibberish” (207; my emphasis). Here space too is no longer seen to be organized according to the subject’s interests. In Merleau-Ponty’s account, the schizophrenic patient can experience the world as visually “murky” (1989, 336); Malone’s air, a “restless gloom”, likewise “grow[s] murky and dim, thickening is perhaps the word, until all things blotted out” (224). Just as for Merleau-Ponty’s schizophrenic the “impulse towards things has lost its energy” (1989, 336), Malone describes his own perceptual inertia: “I am not one of those people who can take in everything at a single glance, but I have to look long and fixedly and give things time to travel the long road that lies between me and them” (238).

These descriptions lose their phenomenological richness to some extent in *The Unnamable*, the final volume, where they gain a greater level of abstraction, but the protagonist of this novel also dwells on the impossibility of perceiving or organizing his world, his being even darker—“this ridiculous black which I thought for a moment worthier than the grey to enfold me”—and he cannot make things meaningful in the face of an overwhelming indifference: “Here there is no wood, nor any stone, or if there is, the facts are there, it’s as if there wasn’t […] I see my place, there is nothing to show it, nothing to distinguish it, from all the other places” (366–67). He too is, like Merleau-Ponty’s schizophrenic subject, “cut off from the common property world” towards which most people’s “existence rushes” (1989, 335). Schizophrenia research has observed a link between anhedonia, an indifference towards both physical experience and social and emotional contact, and the ability to discern relevant (and disengage from irrelevant) stimuli in perceptual tests. Beckett’s exploration of profound indifference as it strikes (or perhaps creeps up on) his protagonists explores its cognitive as well as emotional implications.

For Merleau-Ponty’s patient, time as well as space is perceived differently from those in the “common property world”. Not rushing towards this world, they cannot feel time passing with the same alacrity, just as Malone feels it an age before things can pierce his consciousness and assume an identity (if they do at all). For people with schizophrenia, time may stop or repeat itself, being felt to ‘stutter’ (Cutting 1997; Broome 2005), an experience also strikingly portrayed in Beckett’s fiction and theatre. Vladimir in Beckett’s *Waiting for Godot* famously concludes that rather than Pozzo’s watch being broken, “Time has stopped” (1989, 36), a cosmic joke in the context of this play in which stasis and repetitive days make it hard to discern progression or the change that signals the passing of time. In Beckett’s novels, however, the phenomenology of apparently disordered time perceptions is rendered more richly, exploring the interconnectedness of time and space perception.

Eugène Minkowski’s groundbreaking work on the effects of psychopathology on the ability to perceive and think about space and lived time started with his interest in Bergson, a philosopher whom Beckett also read and whose terms and ideas appear sporadically in his writing. Yann Mével has already observed the similarity between some of Minkowski’s observations on the “schizoid” sensibility and those of Beckett’s narrators: a tendency to organize possessions and space in a geometrically regular fashion; a (related) preoccupation with plans and arbitrary formal structures (2008, 279–80). The similarities are even more widespread than Mével indicates. Like Merleau-Ponty, Minkowski teases out the implications of a “*loss of vital contact with reality* [sic]” (1970, 273). Minkowski seems to invoke here a version of Sass’s operative intentionality that he calls “the fact of ‘me-here-now’ in the life of the individual” (274), an awareness of self-presence that schizophrenia attacks, but which in Minkowski’s view is preserved in other kinds of ‘dementia’, the patient knowing that he or she is “here”, even if he or she cannot identify the location. This foundational awareness, what Minkowski calls “the ‘me-here-now’ “in a naked state”, is something close to Nordau’s *coenaesthesia*. For the schizophrenic, shorn of this awareness, he (in Minkowski’s referent) “*knows* very well where he is but […] doesn’t feel at the place where he is” and “the words “I exist” do not have a precise sense for him” (274).

Beckett’s narrators and characters experience a profound inertia similar to Minkowski’s schizophrenic patient, who has “something ‘immobile’ about [him]”. Malone describes the life of Macmann, an avatar for himself: “He sat and lay down at the least pretext and only rose again when the élan vital or struggle for life began to prod him in the arse again” (244). Beckett here, like Minkowski, uses Bergson’s terms (*élan vital*) for the vital principle that represents not just energy and purpose, but also a perceptual “attention to life”. For the most part, however, Macmann spent:

[…] a good half of his existence […] in a motionlessness akin to that of stone, not to say three quarters […] a motionless at first skin deep but which little by little invaded, I will not say the vital parts, but at least the sensibility and understanding. (244)

Minkowski’s anonymous patient, similarly, observes:

I look for immobility. I tend towards repose and immobilization. I also have in me a tendency to immobilize life around me. Because of this, I love immutable objects, things which are always there and which never change. Stone is immobile. (Anonymous patient quoted in Minkowski 1970, 279)

His account of his life and aspirations seems to be a blueprint for Beckett’s *Trilogy*:

Throughout this day I will try to do nothing at all. I will go for forty-eight hours without urinating. I will try to revive my impressions of fifteen years ago, to make time flow backward, to die with the same impression with which I was born, to make circular movements so as to not move too far away from the base in order not to be uprooted. This is what I wish.

Although some of these elements are there of necessity for Beckett’s infirm narrators, it is striking how similarly they live to the prescriptions of this individual. The last story that Malone tells himself will be one “of a stone”, suggesting as several critics have pointed out the predilection of Beckett’s characters for the inorganic and immobile. His dying seems increasingly to offer similar impressions to his birth, wallowing in a “nourishing murk” (193), the window of his room “in a manner of speaking my umbilicus” (224) and at the “confines of this restless gloom a glimmering as of bones”, in his head “all streaming and emptying away as though a sluice, to my great joy”. This is, as he says, a “deliverance” with various phases. These disparate impressions come into sharp focus a little later in the narrative, when he sees himself explicitly as “an old foetus now” whom his mother will “drop […] with the help of gangrene” (226). The narrator of *The Unnamable* even tells a curious story in which he too makes circular movements, “laps” in a “kind of inverted spiral” around the “base” of his family home in order to “return to the fold”, the “nest [he] should never have left” (320–21).

The convergences between Beckett’s writing and one isolated testimony from a patient prove little, of course, although there is a cumulative effect of reading accounts in which the speakers feel withdrawn from the world not in relation to social anxiety, hostility, or loneliness but as a result of an indifference so great as to affect time and space perception (a “nut in a great and hard shell”, as one patient put it (Minkowski 1970, 284)) and threaten one’s intentionality. These sensations are strikingly similar to moments of indifference—or sudden attachment to unexpected objects—in Beckett’s writing. In his narratives, indifference goes beyond affect to become a structural principle. Perceptions of time and space are not so much disordered as reordered in line with a lack of future time perspective, a saturation of internal space (as Franz Fischer has described schizophrenia quoted in Minkowski 1970, 275) and an experience of depersonalization all characteristic of psychotic disorders (although also found in other conditions). It is very difficult for either clinician or reader of Beckett to have any subjective understanding of what Minkowski’s observation that “the words “I exist” do not have a precise sense for him” could mean. Beckett’s writing may help us to some awareness of this predicament.

That there are similarities between the experience of a series of increasingly abstract fictional narrators and that of real individuals is of course of limited significance in the clinical sphere. It may be, also, that it takes the later theatrical works to give a concrete sense of the phenomenological experience of some of the features discussed. The imaginative exercise that contemplates the attitudes depicted in the *Trilogy* may be predominantly a formal rather than an affective one. There is something suggestive, however, about the concern of Beckett’s work with a level of awareness below the Cartesian consciousness, and a disruption to ipseity that affects cognitive, somatic and ontological aspects of being in the world. The link Beckett draws between these formal disruptions and the condition of indifference, in particular, mirrors in an intriguing fashion the links drawn in schizophrenia research between (negative) anhedonia and both cognitive disorganization and perceptual disturbance. These may not be clinical insights, but they are imaginative possibilities that both clinician and scholar could productively entertain.

## Endnotes

^1^ Beckett also spent some time in a letter to the German translator of *Molloy*, Erich Franzen, on 17 February 1954 explaining this term with reference to the *Oxford English Dictionary*, the pathologist Friedrich Henle and the psychologist Wilhelm Wundt (Beckett 2011, 458–460).
